# Cell cycle checkpoint status in human malignant mesothelioma cell lines: response to gamma radiation

**DOI:** 10.1038/sj.bjc.6600736

**Published:** 2003-02-10

**Authors:** C Vivo, C Lecomte, F Levy, K Leroy, Y Kirova, A Renier, L Kheuang, P Piedbois, D Chopin, M C Jaurand

**Affiliations:** 1 INSERM EMI 9909, Faculté de Médecine, Université Paris XII, rue du Général Sarrail, 94010 Créteil, Cedex, France; 2 EA 2348, Département de Pathologie, Hôpital Henri Mondor, AP-HP, 51 av de Lattre de Tassigny, 94010 Créteil, France; 3 Service d'Oncologie, Hôpital Henri Mondor, AP-HP. 51 av de Lattre de Tassigny, 94010 Créteil, France

**Keywords:** mesothelioma cells, cell cycle arrest, *γ*-radiation

## Abstract

Knowledge of the function of the cell cycle checkpoints in tumour cells may be important to develop treatment strategies for human cancers. The protein p53 is an important factor that regulates cell cycle progression and apoptosis in response to drugs. In human malignant mesothelioma, p53 is generally not mutated, but may be inactivated by SV40 early region T antigen (SV40 Tag). However, the function of p53 has not been investigated in mesothelioma cells. Here, we investigated the function of the cell cycle checkpoints in six human mesothelioma cell lines (HMCLs) by studying the cell distribution in the different phases of the cell cycle by flow cytometry, and expression of cell cycle proteins, p53, p21^WAF1/CIP1^ and p27^KIP1^. In addition, we studied p53 gene mutations and expression of SV40 Tag. After exposure to *γ*-radiation, HMCLs were arrested either in one or both phases of the cell cycle, demonstrating a heterogeneity in cell cycle control. G1 arrest was p21^WAF1/CIP1^- and p53-dependent. Lack of arrest in G1 was not related to p53 mutation or binding to SV40 Tag, except in one HMCL presenting a missense mutation at codon 248. These results may help us to understand mesothelioma and develop new treatments.

Malignant mesothelioma (MM) is an insidious tumour with a dismal prognosis. Owing to past asbestos exposure and because of the long latency period of the disease, mesothelioma mortality will continue to increase over the next decades (Peto *et al*, 1999; Banaei *et al*, 2000). Knowledge of biological features and specific molecular targets of cancer cells may lead to advances in both diagnosis and therapy (Karp and Broder, 1994; Blagosklonny and Pardee, 2001). Cell cycle regulation is of particular interest in the overall control of cancer cell survival. (Shah and Schwartz, 2001). The protein p53 is an important cell cycle regulator that induces expression of genes that halt the cell cycle and trigger cell death (Agarwal *et al*, 1998; May and May, 1999). While p53 is mutated in many types of tumours, mutations were found only in a limited number of MM (Greenblat *et al*, 1994). On the other hand, in MM, p53 may be inactivated via its association with the SV40 early region T antigen (SV40 Tag) (Testa and Giordano, 2001). Thus, strategies have been proposed recently to induce arrest of MM cell proliferation and apoptosis by the restoration of p53 function. This has been carried out following gene transfer, either by the overexpression of p53 (Giuliano *et al*, 2000), by the expression of regulators of p53 levels such as p14^ARF^ (Yang *et al*, 2000) or by the expression of antisense transcripts to Tag (Waheed *et al*, 1999). So far, however, a number of MM have not been found to express SV40 Tag (Strickler *et al*, 1996). Also, these methods may not be functional in cells that do not contain viral molecules, as quoted by Waheed *et al* (1999). For strategies involving anticancer drugs, the effect on cancer cells depends both on the nature of the drug and on the molecular status of the cancer cells, particularly p53 activity (Bunz *et al*, 1999). Thus, it would be important to determine whether the function of p53 is inactivated in MM cells and to investigate their response to p53 activating agents. Therefore, we studied the activity of p53 in human MM cell lines (HMCLs) by characterising the function of the G1/S and G2/M checkpoints in six HMCLs exposed to *γ*-radiation, and by the determination of their associated p53 mutation status and Tag SV40 expression.

We found that all cell lines were responsive to irradiation, as shown by cell cycle arrest, but two HMCLs failed to arrest in G1 phase of the cell cycle. No apoptosis was observed. Arrest in G1 was p21^WAF1/CIP1^-dependent via p53. No SV40 Tag expression was found, but one HMCL presented a missense mutation at codon 248. These results demonstrate that HMCLs appear to be resistant to DNA-damaging agents and suggest that therapies combining abrogation of cell cycle checkpoints and enhancement of the cell death mechanisms should be investigated in MM.

## MATERIALS AND METHODS

### Human mesothelioma cell lines (HMCLs)

Human mesothelioma cell lines were obtained from confirmed MM cases and were cultured as described elsewhere (Buard *et al*, 1998) in RPMI 1640 medium with L-glutamine supplemented with 8% fetal bovine serum, 10 mmol l^−1^ HEPES buffer (both from Life Technologies, Inc., Cergy Pontoise, France), 50 U ml^−1^ penicillin and 50 *μ*g ml^−1^ streptomycin (ATGC Biotechnologie, Noisy le Grand, France).

Six HMCLs, RV, BT, BR, BL, CR and FR obtained from mesothelioma cases were used in the present study. They were used between passages 3 and 15. The mesothelial origin of the cells was assessed by the coexpression of cytokeratin and vimentin and absence of expression of carcinoembryonic antigen (Zeng *et al*, 1994). Moreover, an expression of calretinin was found in all HMCLs using the rabbit polyclonal anticalretinin antibody (Zymed) (unpublished data).

### Treatment with *γ*-radiation

Cells were plated on 75 cm^2^ flask (Costar, Dutscher, Brumath, France) at 2×10^4^ cells cm^−2^. At 70% confluence, cells were washed with phosphate-buffered saline (PBS) 1×, and flasks were filled to the neck with serum-free RPMI 1640 culture medium. HMCLs were irradiated at 6, 9 or 12 Gy (1.2 Gy min^−1^) by exposure to a ^60^Co source (Alcyon from General Electric Medical System, France). After irradiation, the serum-free RPMI was replaced with complete RPMI 1640 medium and the flasks were returned to 37°C.

### Cell cycle analysis by flow cytometry

After different durations of exposure to *γ*-radiation, bromodeoxy-uridine (BrdU) (Sigma, St Quentin-Fallavier, France; final concentration: 18 *μ*g ml^−1^) was added to the culture medium for 1 h. After incubation, the cells were treated as described elsewhere (Vivo *et al*, 2001). Briefly, cells were trypsinised, washed in PBS, fixed in 70% ethanol and resuspended in 2N HCl at room temperature for 30 min. After washing with 0.5% Tween 20 in PBS (PBST), cells were centrifuged and rinsed until the pH settled between 7.2 and 7.4, and incubated with BrdU antibodies diluted 1 : 10 in PBST (Dako, Trappes, France) at room temperature for 30 min, followed by three washes with PBST. The pelleted cells were then resuspended and incubated 30 min at room temperature in 100 *μ*l of anti-mouse IgG antibodies (Dako, Trappes, France) conjugated with fluorescein isothiocyanate (FITC), diluted at 1 : 20 in PBST. After three washes, cells were stained with propidium iodide (PI) (0.05 mg ml^−1^ final concentration) for 1 h at room temperature in the dark. As a control for FITC specificity, the first antibody was omitted from the incubation procedure in some samples.

In total, 30 000 HM cells were analysed per treatment with a flow cytometer Coulter Epics XL WO5039 (Coultronics, Margency, France). A dual parameter histogram of the cell cycle phase distribution and calculations of the percentage of cells in G0/G1, S and G2/M phases were obtained by analysis of each sample using XL software. Distribution of cells in the different phases of the cell cycle was also analysed by Multicycle software (Phoenix Flow Systems, San Diego, CA, USA). Each experiment was performed in triplicate. Data are presented as a representative sample.

### PCR and RT–PCR analyses of SV40 Tag DNA sequences

Cell DNA was amplified using the primers PYV. for (5′-TAGGTGCCAACCTATGGAACAGA-3′) and PYV. rev (5′-GGAAAGTCTTTAGGGTCTTCTACC-3′) (Carbone *et al*, 1994). Polymerase chain reaction (PCR) was carried out with Taq DNA polymerase (Invitrogen). Thermocyclin was performed by denaturation at 94°C for 3 min, followed by cycling 35 times at 94°C for 1 min, at 55 for 1 min, and at 72°C for 1 min. The products were separated by electrophoresis. As positive control, SV40 Tag transduced rat pleural mesothelial cells were used (Pilatte *et al*, 2000).

RNA was extracted from cell lines using the RNeasy Mini kit (Qiagen). Total RNA (1.5 *μ*g) was reverse transcribed with oligodT (Promega) using the Superscript II RT kit (Gibco BRL) following the manufacturer's recommendations. PCR was performed with PYV.for and PYV.rev primers. A measure of 2 *μ*l of the cDNA reaction product was amplified in 1× buffer, 0.2 mM dNTPs, 1.5 mM MgCl_2_, 0.25 *μ*M primers and 25 mU *μ*l^−1^ *Taq* DNA polymerase (Invitrogen). After an initial denaturation at 94°C for 2 min, 35 cycles were performed consisting of denaturation at 94°C for 30 s, annealing at 55°C for 1 min and extension at 72°C for 1 min. The final extension step was continued for 5 min. PCR product was analysed by electrophoresis on a 2% agarose gel and ethidium bromide staining. GAPDH amplification (quantitative control) was performed as described above with GAPDH5′ (5′-GGATTTGGTCGTATTGGGCGC-3′) and GAPDH3′ (5′-GTTCTCAGCCTTGACGGTGC-3′) primers.

### Protein analysis by Western blot

#### Total cell extracts

To prepare extracts, the cell layer was washed three times with 10 ml PBS and scraped on ice after addition of a lysis buffer (1 mM EDTA, 5 mM NaF, 1% NP40, 1 mM sodium orthovanadate, 1 *μ*g ml^−1^ leupeptine, 25 *μ*M NPGB and 5 mM sodium pyrophosphate in PBS). Cells were further lysed by repeated passages through a 21-gauge needle, and lysates were cleared by centrifugation at 16 000 **g** for 15 min at 4°C.

#### Nuclear and cytoplasmic protein extraction

After washing in PBS, cells were scraped on ice in a sucrose buffer (250 mM sucrose, 3 mM imidazole and 1 mM EDTA used as protease inhibitor) in PBS 1X, lysed by repeated passages through a needle. After centrifugation at 300 **g** for 10 min at 4°C, the cytoplasmic protein concentration of the supernatant was determined. The pellet (nuclei and unbroken cells) was resuspended in sucrose buffer, homogenised with needle as above and centrifuged at 300 **g** for 10 min. Both supernatants (cytoplasmic fraction) were pooled and centrifuged at 100 000 **g** for 1 h. The pellet was resuspended in lysis buffer (see above ‘Total cell extracts’), homogenised with needle and centrifuged at 15 000 **g** for 15 min at 4°C. This supernatant contained the nuclear fraction.

#### Immunoprecipitation for SV40 Tag detection

SV40 Tag detection was performed as described elsewhere (Pilatte *et al*, 2000). Briefly, MM cells were washed, scraped in lysis buffer (see above ‘Total cell extracts’) and lysed by repeated passages through a needle. Lysates were cleared by centrifugation at 16 000 **g** for 15 min at 4°C. After preclearing with Protein A–Sepharose beads (Pharmacia Biotech, Orsay, France), protein concentration was determined using the DC Protein assay (Bio-Rad, Ivry sur Seine, France) and normalised by addition of extraction buffer. The cell lysate was incubated with anti-p53 antibody (D01, Santa Cruz, Biotechnology, Inc., Tebu, France, Le Perray-en-Yvelines) for 2 h at 4°C (3 *μ*g antibody: 1 mg total pro-tein). Immunocomplexes were collected on protein A–Sepharose 1 h at 4°C, and washed four times with immunoprecipitation buffer (40 mM HEPES, 8 mM MgCl_2_, 100 mM NaCl, 0.5% Nonidet P-40, 2 *μ*g ml^−1^ aprotinin, 2 *μ*g ml^−1^ leupeptin, 100 *μ*M Na_3_VO_4_). Immunocomplexes were resuspended in loading buffer and analysed by Western blot using anti-SV40 Tag antibody (Pab 101, Santa Cruz). Positive control consisted in a cell extract from SV40 Tag transduced rat pleural mesothelial cells (Pilatte *et al*, 2000).

#### Western blots

The following antibodies were used: primary mouse monoclonal antibodies against human p21^WAF1/CIP1^, p27^KIP1^, p53 (D01) and SV40 Tag (Ab-1), all from Santa Cruz.

A total of 40 *μ*g of total protein extract was boiled for 5 min in SDS loading buffer with 1% *β* mercaptoethanol, and loaded onto 7.5–12% SDS-polyacrylamide gel(PAGE), depending on the protein type. After electrophoresis, proteins were transferred to an Immobilon-P membrane (Millipore, St Quentin en Yvelines, France). The protein transfer was confirmed with a red Ponceau staining and the membrane was blocked with 5% nonfat milk in PBST for 1 h at room temperature. Peroxidase-conjugated anti-mouse IgG secondary antibody (Santa Cruz) was used at 1 : 2000 dilution in PBS containing 5% nonfat milk and 0.5% Tween 20 for 1 h at room temperature and washed again for 30 min in PBS. Proteins were detected using the ECL method (Amersham, Life Science, Les Ulis, France).

### p53 mutation analysis

#### Amplification by PCR

Genomic DNA was isolated from HMCLs and normal lymphocytes by proteinase K digestion and phenol/chloroform extraction. The DNA (100 ng) was amplified (Perkin-Elmer). Primers to amplified fragments of the p53 gene (Table 1

**Table 1:** Sequences of P53 primers

**Amplified fragment**	**Nucleotide position in genomic DNA**	**Fragment size (bp)**		**Primers^a^**
Exon 2	11 642–11 873	232	(1)	CAGGGTTGGAAGCGTCTCATG
			(2)	AATTTTCGCTTCCCACAGG
Exon 3	11 832–11 951	120	(1)	CAACCCCAGCCCCCTAGCAG
			(2)	TCCCAGCCCAACCCTTGTCC
Exon 4	11 925–12 416	492	(1)	CTGGTAAGGACAAGGGTTGG
			(2)	ACACTGACAGGAAGCCTAAGGGT
Exon 5	12 969–13 328	360	(1)	TTTGTTTCTTTGCTGCCGTGTT
			(2)	GGGCCAGACCTAAGAGCAATCA
Exon 6	13 246–13 564	319	(1)	GCTGGGGCTGGAGAGACGACA
			(2)	ACTTTGCACATCTCATGGGGTT
Exon 7	13 943–14 378	436	(1)	TGCCACAGGTCTCCCCAAGG
			(2)	GGCTCCATCTACTCCCAACCACC
Exon 8	14 373–14 616	244	(1)	GGAGCCTGGTTTTTTAAATGGG
			(2)	TCCACCGCTTCTTGTCCTGCT
Exon 9	14 596–14 849	254	(1)	AGCAGGACAAGAAGCGGTGGA
			(2)	GCCCCAATTGCAGGTAAAACA
Exon 10	17 543–17 735	193	(1)	TTACTTCTCCCCCTCCTCTGTTG
			(2)	GCTTTCCAACCTAGGAAGGCAG
Exon 11	18 567–18 795	229	(1)	TCATCTCTCCTCCCTGCTTCTGTC
			(2)	TGCTTCTGACGCACACCTATTG

a (1) sense primer; (2) antisense primer.

) from exons 2–9 were selected according to previous research in the laboratory and primers for exons 10 and 11 were selected from previously published information (Kazachkov *et al*, 1996). Amplification of exons 2–9 was performed in two steps: first, a multiplex PCR was used to amplify exons 2–9 in three parts (exons 2–4; exons 5 and 6; exons 7–9) and second, a nested PCR was used to amplify for each exon. Multiplex PCR cycle amplification for exons 2–9 was 1 cycle for 5 min, 92°C; 30 cycles for 90 s, 92°C; 105 s, 58°C; 150 s, 72°C and 1 cycle for 15 min, 72°C. Nested PCR cycle amplification for exons 2–9 was 1 cycle for 5 min, 92°C; 30 cycles for 90 s, 92°C; 105 s, 54°C; 150 s, 72°C and 1 cycle for 15 min, 72°C. Exons 10 and 11 amplification was performed directly in nested PCR. The amplification cycle for exon 10 was 7 min, 94°C; 40 cycles, 1 min, 95°C; 1 min, 58°C; 1 min, 72°C and 7 min, 72°C. The amplification cycle for exon 11 was 7 min, 94°C; 35 cycles, 1 min, 95°C; 1 min, 55°C; 1 min, 72°C and 7 min, 72°C.

#### Single-strand conformation polymorphism (SSCP)

SSCP analysis was performed on each PCR product for exons 2–9. Each PCR product was mixed with one volume of denaturating solution (95% formamide, 20 mM EDTA, 0.05% xylene cyanol, 0.05% bromophenol blue), heated for 5 min at 95°C, cooled on ice and subjected to nondenaturing electrophoresis in a 10% PAGE (Clean gel 48S kit, Amersham Pharmacia Biotech, Sweden). The gels were electrophoresed for 1 h at 200–600 V at 15°C for exons 2–8 and 4°C for exon 9. After migration, gels were treated with the Plusone kit for silver DNA staining (Pharmacia Biotech) and dried.

#### DNA sequencing

DNA sequencing was performed on any PCR product that showed a conformational change in SSCP analysis and on all the PCR products of exons 10 and 11. Nucleotide sequences were determined by the Dye terminator method for exon 4 (ABI PRISM Dye Terminator Cycle Sequencing Ready Reaction Kit with AmpliTaq DNA Polymerase, FS, Perkin-Elmer) and Dye Primer ready reaction mixes for exon 9. Fragments were sequenced on both strands. Sequencing reactions were loaded on a 4.25% acrylamide–7 M urea denaturing gel using an ABI 377 (Applied Biosystems, Foster City, CA, USA) sequencing apparatus and mutational analysis performed with ‘Sequence Navigator’ Software. PCR products of exons 10 and 11 were sequenced by Eurogenteck (Belgium). Computer analysis of the mutations was performed with ‘Sequence Navigator’ software.

## RESULTS

### Cell distribution in the different phases of the cell cycle after exposure to *γ*-radiation

**Figure 1:**
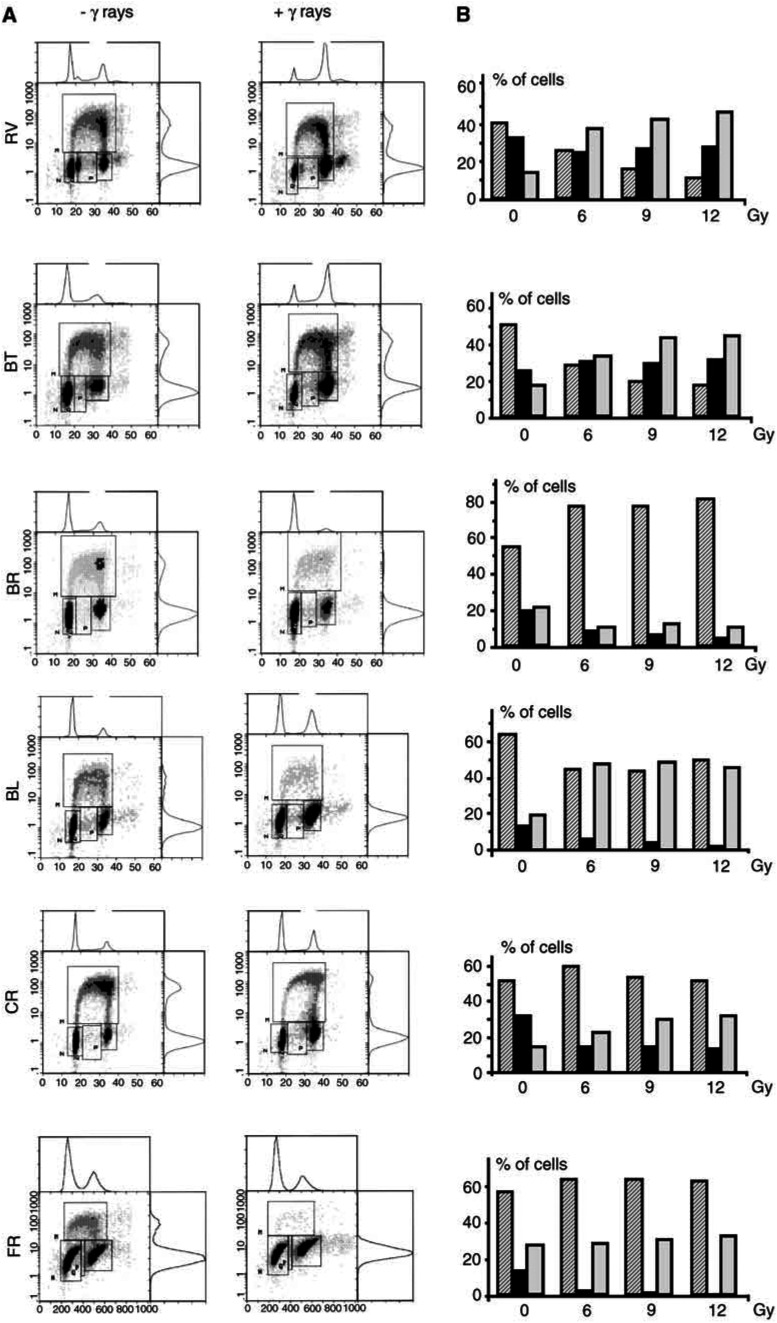
Flow cytometric analysis of HMCLs after exposure to *γ*-radiation. (**A**) Left: nonirradiated cells; right: 24 h after exposure to 6 Gy. Double labelling: PI (*x*-axis) and BrdU (*y*-axis). The different areas N, M and P represent cells in G0/G1, cycling S cells and cells in G2-M, respectively. (**B**) Dose-dependent cell distribution in different phases of the cell cycle. Percentage of cells in G0/G1 (hatched bars), S (black bars), G2/M (grey bars), 24 h after exposure to 6, 9 and 12 Gy.

Two cell lines, RV and BT, did not arrest in G1 phase (2C amount of DNA), since a concurrent decrease of the percentage of cells in G0/G1 phase was found, as well as a nearly unmodified BrdU incorporation (Figure 1A

). However, these two cell lines exhibited an arrest in G2/M phase, as demonstrated by the marked increase in the number of cells with a 4C DNA content on the PI profile. No subG1 peak of apoptotic cells was observed. Histograms (Figure 1B) show that the enhancement in the percentage of cells arrested in the G2/M phase was dose-dependent.

In contrast, four HMCLs presented an arrest in G1 phase after similar exposure but also with no apoptosis. G1 arrest is demonstrated in the cell line BR, by an increase in the percentage of cells present in the G0/G1 compartment and a decrease in cells labelled with BrdU. Moreover, a decrease in the number of cells corresponding to the 4C amount of DNA was observed, in agreement with a lack of G2/M arrest in this cell line. The histogram (Figure 1B) shows that these effects were dose-dependent. Dot plot and histogram analyses of the other cell lines (BL, CR and FR) suggested an arrest both in G0/G1 and G2/M phases of the cell cycle after exposure to *γ*-radiation. All three cell lines exhibited a strong decrease in the number of cells incorporating BrdU in response to *γ*-radiation, in agreement with a G1 arrest; this was associated with a net increase of the amount of cells in the G2/M compartment in the CR and BR cell lines. In the FR cell line, the arrest in G1/S is suggested by the lack of decrease in the proportion of cells present in the G1 compartment, after irradiation.

**Figure 2:**
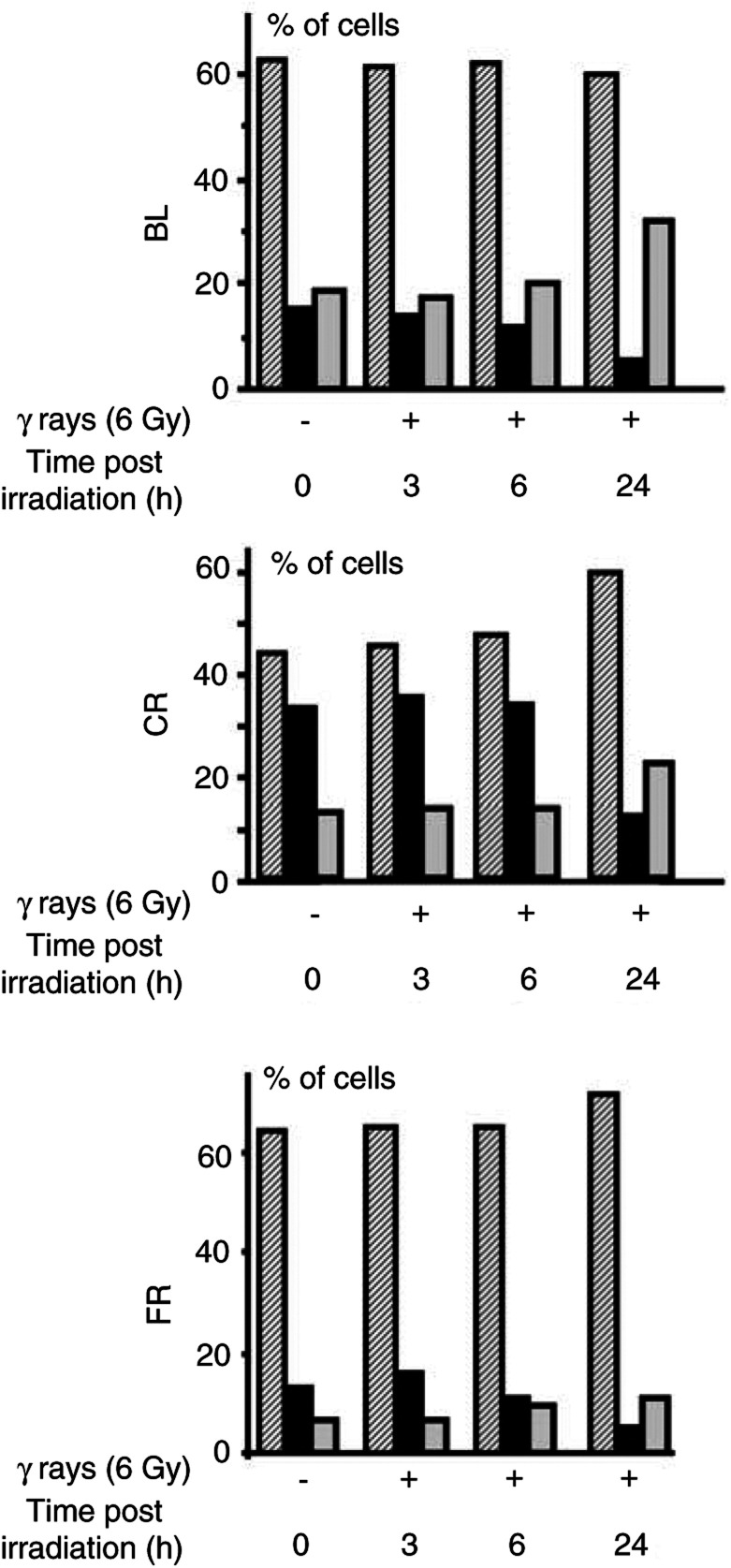
Kinetics of the distribution of mesothelioma cells (BL, CR and FR HMCLs) in the different phases of the cell cycle: G0/G1 (hatched bars), S (black bars), G2/M (grey bars), at different times after irradiation (6 Gy).

In order to determine the time-dependent response following exposure to *γ*-radiation and to confirm the double arrest in the three cell lines BL, CR and FR, a kinetic study of the cell distribution in the phases of the cell cycle was carried out after exposure to 6 Gy. Accordingly, the results show a time-dependent decrease in the percentage of cells in S phase (Figure 2

), associated with an increase or a stabilisation in the percentage of cells in G1 and G2/M phases. Modifications of the cell distribution were detectable from different time postirradiation depending on the cell line.

#### p53 expression in HMCLs exposed to *γ*-radiation

**Figure 3:**
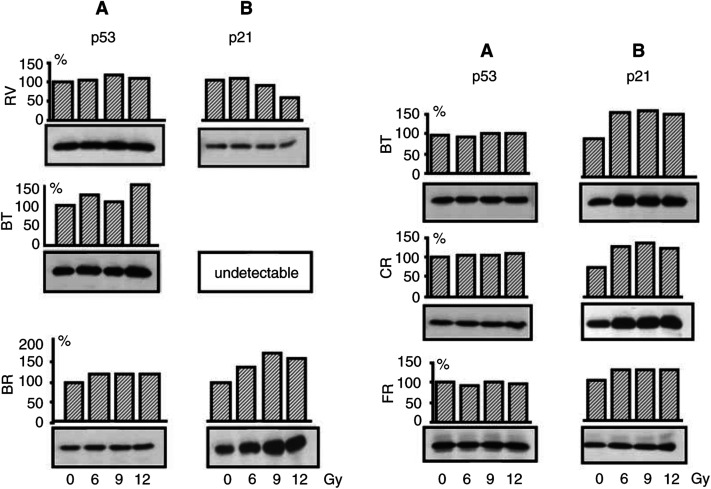
p53 (**A**) and p21^WAF1/CIP1^ (**B**) protein expression in HMCLs after exposure to several doses of *γ*-radiation. At 24 h after irradiation, protein extracts were subjected to SDS-PAGE electrophoresis followed by immunoblot analysis with antibodies against the corresponding antigens. ECL detection. Densitometric analyses of p53 and p21^WAF1/CIP1^ expression are reported on the top of the corresponding bands as percentage of the amount of protein expressed in untreated cells.

**Figure 4:**
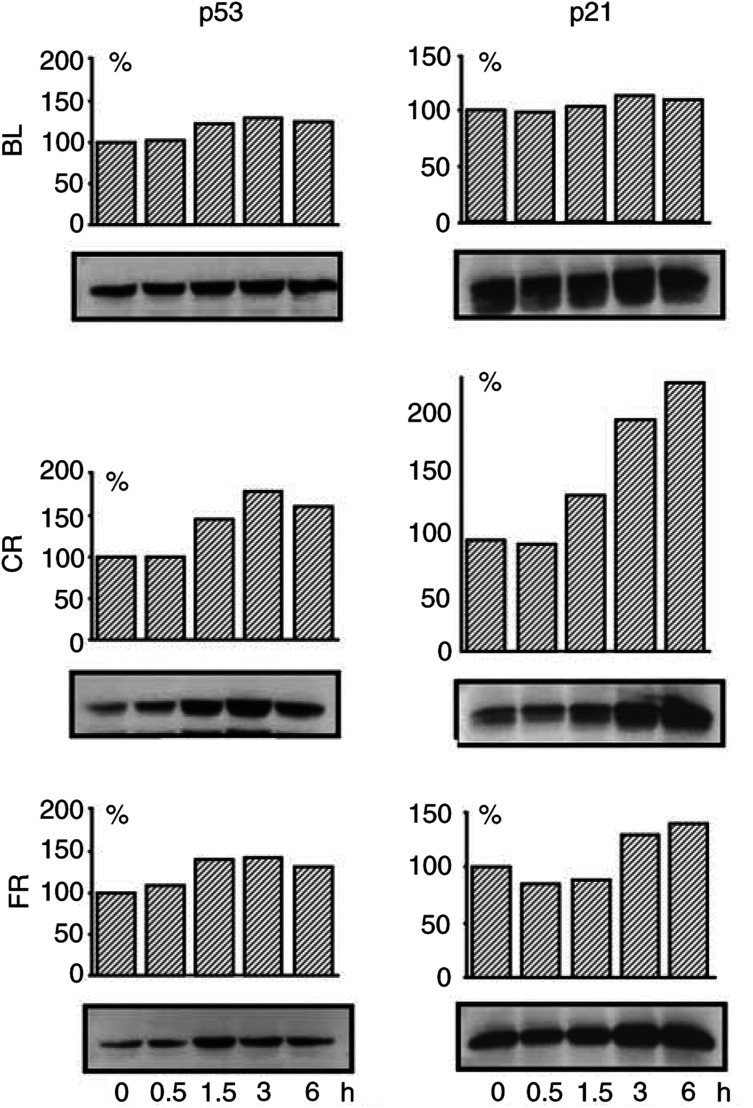
Kinetics of p53 and p21^WAF1/CIP1^ expression in HMCLs (BL, CR and FR) showing an arrest at the G1/S transition in response to *γ*-radiation. Cells were exposed to 6 Gy as described in Materials and Methods and protein extracts were analysed at the indicated times postirradiation. Densitometric analyses are expressed as in Figure 3.

HMCLs were treated according to the same protocol as for the cell cycle studies. Western blot analysis of cells exposed to different doses of radiation showed no evident modification in the expression of p53 protein in the majority of HMCLs 24 h after irradiation (Figure 3A

). However, at an earlier time point (0–6 h after irradiation) an enhancement of p53 protein expression was observed in cell lines showing an arrest in G1 suggesting a transient stabilisation of p53 in these HMCLs (Figure 4

).

#### Subcellular localisation of the p53 protein

Since p53 is a transcription factor, its subcellular localisation is of importance to account for its activity. The intracellular localisation of p53 was investigated in five HMCLs following exposure to 6 Gy, after concentration of nuclear proteins as described in Materials and Methods. The results indicate that p53 was present in the nuclear fraction of all HMCLs both in the absence of treatment and after irradiation (data not shown).

#### Effect of exposure to *γ*-radiation on p21^WAF1/CIP1^ and p27^KIP1^ expression in HMCLs

In the HMCLs failing to arrest at the G1/S transition, no induction of p21^WAF1/CIP1^ was found; instead, p21^WAF1/CIP1^ protein decreased in RV and p21^WAF1/CIP1^ was undetectable in BT (Figure 3B). In contrast, in cells with a G1 arrest, p21^WAF1/CIP1^ expression was enhanced at different times postirradiation. After 24 h, a clear induction was observed in BR, BL and CR at all experimental doses and moderately in FR (Figure 3B). However, at earlier time points, 1.5 and 3 h, an induction was found in CR and FR cell lines (Figure 4).

After irradiation, p27^KIP1^ expression was constant in HMCLs exhibiting a double arrest in G1 and G2/M phases (BL, CR and FR). p27^KIP1^ protein expression was markedly lowered in a dose-dependent manner in the HMCLs that did not arrest in G1 (RV and BT) (data not shown). In contrast, a dose-dependent enhancement in p27^KIP1^ was found in BR, a cell line that exhibited a G1 blockade not associated with a G2/M arrest.

#### SV40 Tag DNA and RNA sequence detection and protein expression

**Figure 5:**
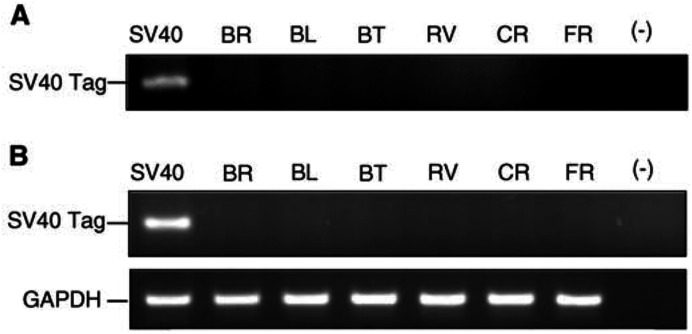
PCR (**A**) and RT–PCR (**B**) analyses of DNA and RNA extracted from HMCLs as described in Materials and Methods. Amplifications were carried out with PYV.for and PYV.rev for SV40 Tag primers. Positive control was SV40 Tag transduced rat pleural mesothelial cells (SV40). (−): negative control. GAPDH amplification (165 bp) was used as quantitative RT–PCR control.

**Figure 6:**
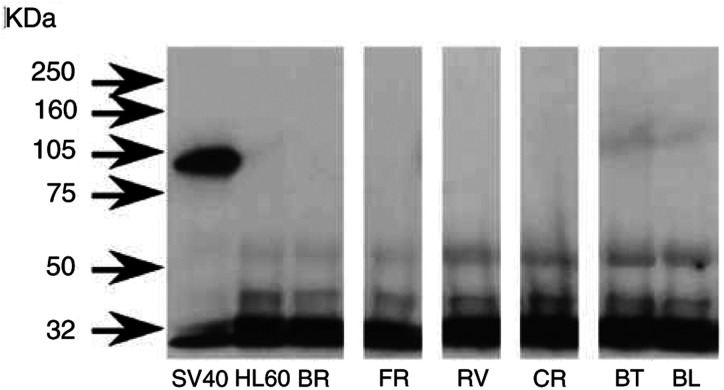
Western blot analysis of SV40 Tag. Cell extracts were immunoprecipitated with an anti-p53 antibody and the resulting immunoprecipitates were immunobloted with anti-Tag antibody as described in Materials and Methods. None of the six HMCLs investigated here and HL60 (negative control) presented a band corresponding to SV40 Tag, whereas the positive control (SV40) showed the expected band at 94 kDa.

SV40 Tag DNA and RNA sequences were not detected by PCR except in the positive control (Figure 5

). By Western blot analysis, neither the HMCLs investigated here nor the HL60 line (negative control) exhibited a band corresponding to SV40 Tag, whereas the positive control presented the expected band at 94 kDa (Figure 6

).

#### Investigation of P53 mutation

**Figure 7:**
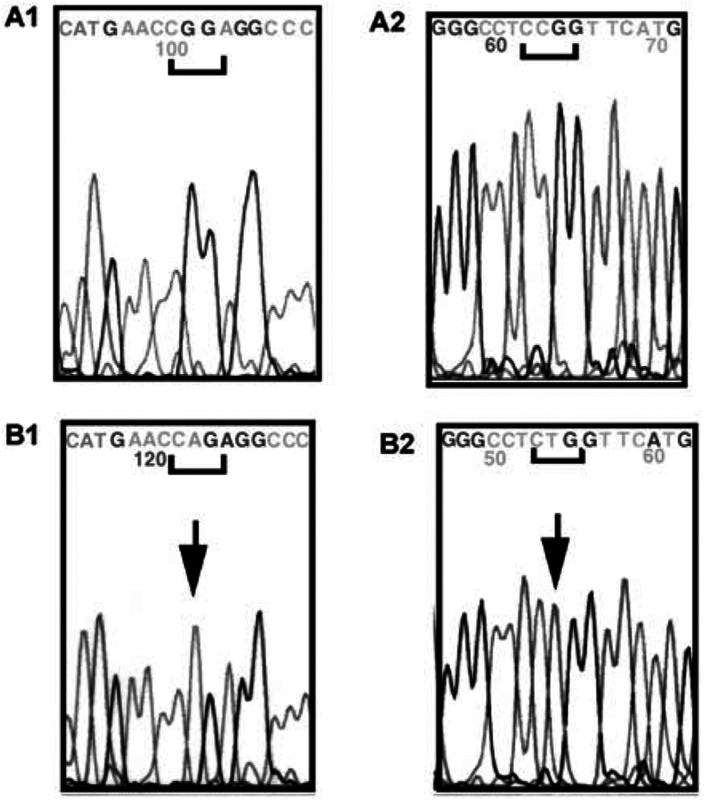
Electrophoregram of *P53* gene part of exon 7: (**A1**) coding strand of CR cell line; (**A2**) noncoding strand of CR cell line. (**B1**) coding strand of BT cell line; (**B2**) coding strand of BT-cell line.

Results showed that one cell line (BT) exhibited a *P53* gene mutation. This mutation is a transition affecting codon 248 (CGG→CAG) converting an arginine into glutamine associated with a loss of the wild-type allele (Figure 7

).

## DISCUSSION

In this study, we investigated cell cyle control in HMCLs exposed to *γ*-radiation by flow cytometry, and characterised their p53 status through analysis of p53 mutation status and Tag SV40 expression.

Our results demonstrate that four of six HMCLs responded to *γ*-irradiation by cell cycle arrest in G1 phase. The two HMCLs that failed to arrest in G1 were arrested in G2/M. G1 arrest in HMCLs appears to be p53- and p21^WAF1/CIP1^-dependent, as shown by the dose-dependent enhancement of p21^WAF1/CIP1^ expression in the G1-arrested cell lines. These results agree with cited experiments demonstrating that p21^WAF1/CIP1^ is necessary for p53-mediated G1 arrest in human colon carcinoma cells generated by DNA-damaging agents (Waldman *et al*, 1995). Interestingly, the strongest enhancement of p21^WAF1/CIP1^ was found in an HMCL that arrested only in G1 (BR). In contrast, two HMCLs that did not respond to irradiation with enhancement of p21^WAF1/CIP1^ expression did not arrest in G1.

The inhibitor of the cyclin E- (cyclin-dependent kinase) (CDK2) p27^KIP1^, of which accumulation is observed in response to antiproliferative signals, does not appear to be involved in the response of MM cells to *γ*-radiation. An enhancement of p27^KIP1^ protein expression was found only in the BR cell line that arrested in G1 (data not shown). This result agrees with the literature findings showing that p27^KIP1^ degradation, because of the phosphorylation and ubiquitination of the protein, occurs in cells that are not in G1 phase (Desdouets *et al*, 2000).

Our results show that all but one HMCL presented a wild-type *P53* gene. A missense mutation at codon 248 was observed in the BT cell line. We believe that this is the first report of such a point mutation in a characterised human mesothelioma cell line. This cell line was obtained from a confirmed human mesothelioma case and exhibited both the ultrastructural features and immunocytochemical characteristics of mesothelial cells based on coexpression of cytokeratin and vimentin, and absence of carcinoembryonic antigen expression (Zeng *et al*, 1994; Fleury-Feith *et al*, 1995). More recent markers, calretinin and CAK1 were also expressed in this cell line. In the literature, this mutation was also reported in a ‘NOS’ (not otherwise specified) mesothelioma cell line (Olivier *et al*, 2002).

It is generally accepted that p53 is required for the G1 arrest of mammalian cells after exposure to *γ*-radiation. We found that p53 was active in four cell lines, but failed to control cell cycle in two HMCLs. p53 is likely to be inactivated by mutation in BT, but no mutation was found in RV. In this cell line, p53 does not appear to be inactivated by Tag SV40, since we were unable to detect Tag SV40 DNA or RNA sequences nor to immunoprecipitate Tag SV40 in the HMCLs investigated here, even though the protein was detected in positive control cells. This is consistent with results reported in other HMCLs (Pilatte *et al*, 2000).

Basal expression of p53 was found in the HMCLs investigated here. Similar findings have been reported in other cancer cell lines (Nagasawa *et al*, 1998; Ashcroft *et al*, 2000). Interestingly, both wild-type p53 and mutant p53^248^ were present in the nucleus as well as in the cytoplasm as determined by Western blotting, but only the mutant protein was detected by immunocytochemistry using antibodies recognising both forms of p53 (data not shown). This suggests strong stabilisation of the mutant p53^248^ in these cells. p53 WT stabilisation in HMCLs is likely associated with deregulation of p53 expression. This may be because of several mechanisms including alteration of mdm2 activity (Blattner *et al*, 2002) and/or of ARF expression (Oren *et al*, 2002). The mechanism by which WT p53 is stabilised in human mesothelioma cells remains to be discovered, but it may be suggested that deregulation of expression is a parameter to consider in the mechanism of mesothelial cell transformation. Alternatively, other tumor suppressor genes *P16/CDKN2A* and *NF2* have been found to play a role in mesothelial oncogenesis (Murthy and Testa, 1999).

Most mammalian cells arrest in G1 and G2 phases of the cell cycle after exposure to *γ*-radiation (Hartwell *et al*, 1994). The role of p53 in G2 arrest is more controversial, likely depending on the type of cell and its biological status. p53 appears to be involved in G2 arrest duration (Bunz *et al*, 1998). Arrest in G2 after DNA damage occurs in the absence of p21^WAF1/CIP1^ and p53 (Waldman *et al*, 1995). Abrogation of p53 by E6 transfection results in loss of G1 control, but not G2 in human colon carcinoma cells (Wouters *et al*, 1999). Our results are consistent with these observations since HMCLs defective in p53 either by mutation (BT) or by failure of p21^WAF1/CIP1^ induction (RV) arrested in the G2/M phase. The present data suggest that the G2 control checkpoint induced by DNA damage is functional in most HMCLs.

In the present experiments, p53 does not appear to trigger apoptosis following *γ*-radiation of HMCLs. From another source, in HMCLs with p53 deficiency, an enhancement of the amount of p53 by transfection with an adenovirus carrying human p14(ARF) cDNA has been found to reduce cell proliferation and enhance apoptosis (Yang *et al*, 2000). Lack of apoptosis in cells with functional p53 may result from regulation by antiapoptotic signals that control cell survival. The role of these factors in the response to drug combination merits further investigation. In the same vein, recent reports show significant augmentation of proapoptotic gene therapy by pharmacological downregulation of survival signals in MM cells (Cao *et al*, 2001; Mohiuddin *et al*, 2001). Furthermore, both tumour necrosis factor-related apoptosis-inducing ligand and chemotherapy cooperate to induce apoptosis in HMCLs (Liu *et al*, 2001).

In conclusion, HMCLs exhibited different responses to *γ*-radiation exposure. However, all cell lines showed activation of the cell cycle control checkpoints after irradiation in either one or in both phases of the cell cycle. The protein p53 may exert a control of cell proliferation in several cell lines, but some failed to activate the p53 function. Our results confirm previous findings of a low mutation rate of p53 in MM and suggest that understanding the details of G1/S and G2/M control checkpoints in mesothelioma cell lines may prove critical to the development of potential novel therapeutic strategies, especially based on the triggering of apoptosis.
